# Racial and ethnic disparities in COVID-19 diagnosis and adherence to mitigation behaviours in a national United States older adult sample

**DOI:** 10.1017/S0950268823001607

**Published:** 2023-10-06

**Authors:** Roger Wong, Margaret Anne Lovier

**Affiliations:** 1 Department of Public Health and Preventive Medicine, Norton College of Medicine, SUNY Upstate Medical University, Syracuse, NY, USA; 2 Department of Geriatrics, SUNY Upstate Medical University, Syracuse, NY, USA

**Keywords:** COVID-19, handwash, mask, race, social distance

## Abstract

Older adults and people of colour are vulnerable to the COVID-19 pandemic, and mitigation behaviours reduce COVID-19 infection. We examined racial and ethnic differences in COVID-19 diagnosis and adherence to COVID-19 mitigation behaviours among U.S. older adults. Data were retrieved from the National Health and Aging Trends Study, a nationally representative prospective cohort with 3257 U.S. Medicare beneficiaries aged 65+. COVID-19 variables were collected in 2020; all other data in 2019. Odds of COVID-19 diagnosis and adherence to mitigation behaviours (handwashing, masking, social distancing) were analysed using logistic regression. Compared to White older adults, only Hispanic respondents had 2.7 times significantly higher odds of COVID-19 after adjusting for sociodemographics, health, and mitigation behaviours (aOR = 2.71, 95% CI = 1.20-6.12). Black older adults had 7.9 times significantly higher odds of masking (aOR = 7.94, 95% CI = 2.33-27.04) and 2.3 times higher odds of social distancing (aOR = 2.33, 95% CI = 1.28-4.24), after adjusting for sociodemographics and health. Among all racial and ethnic groups, only Hispanic older adults had a significantly elevated COVID-19 diagnosis. Despite higher adherence to COVID-19 mitigation behaviours among racial and ethnic minorities, especially Black older adults, odds of COVID-19 remained elevated. Research is needed to explore potential mechanisms for higher odds of COVID-19 among minority older adults.

## Introduction

SARS-CoV-2 has been responsible for the COVID-19 pandemic that has swept across the globe since its emergence in 2019 [1]. As of June 2023, over 6 million patients have been hospitalised with the disease in the United States, and over one million individuals have died [2].

Although anyone can contract and be symptomatic for COVID-19, previous research indicates that COVID-19 does not affect all Americans equally [3]. Age has been found to be one of the greatest risk factors for severe cases and poor health outcomes [4, 5], with adults aged 85 and older having 15 times higher rate of COVID-19 hospitalisation compared with adults aged 18–29 [6]. Immunosenescence may play a role in this finding [4, 5, 7]. Older adults also have higher likelihoods of common COVID-19 comorbidities [7] and, due to those comorbidities, of entering healthcare settings where viral transmission can occur [8, 9]. Records of SARS-CoV-2 infections additionally show that people of colour, particularly those of American Indian or Alaskan Native, Hispanic, or African American heritage, have been especially impacted by COVID-19 morbidity and mortality [8, 10–14]. Pre-existing conditions and socio-economic factors such as essential worker status, limited healthcare access, and cramped, low-resource living conditions likely contribute to these racial and ethnic disparities [8, 10–12, 15].

After the emergence of COVID-19 in the United States in January 2020 [16], several mitigation behaviours have been recommended to reduce the viral spread. Foremost among these have been handwashing (including hand sanitiser usage and other forms of hand hygiene), masking, and social distancing – three behaviours individuals can use to protect themselves and others from contracting COVID-19. However, there have been some hesitancy and resistance to these behaviours across the Western Hemisphere, and not all people have followed state and national guidelines [17–19].

Some of the variance in adherence to COVID-19 mitigation behaviours has fallen along racial and ethnic lines, although findings so far have been at times contradictory. One survey found that Hispanic Americans washed their hands the most frequently out of all major U.S. racial and ethnic groups [17], but others have determined that non-Hispanic White and Asian individuals had the highest odds of practising hand hygiene [18, 20]. Another showed that Black, Hispanic, and Asian Americans have higher odds of wearing masks than Whites [21]. White and Asian or Pacific Islander women were more likely to stay in their homes than other women [21], and African Americans reported that they were less likely to stay indoors [17]. Factors involved in these racial and ethnic differences in following guidelines for mitigation behaviours may relate to an inability to work remotely or otherwise self-isolate, lower health literacy and health literature accessibility, and, in the specific case of hand hygiene, price barriers to purchasing hand sanitiser among minorities [17, 18, 20–22]. It is also possible that racial and ethnic COVID-19 disparities themselves could itself contribute to reduced political support for mitigation behaviours, as White policymakers may be less inclined to combat COVID-19 if they do not believe it is as much a threat to themselves [23]. This can create a downward spiral of even greater COVID-19 spread and inequity [23].

Findings so far suggest that protective behaviours such as social distancing can reduce COVID-19 infection [24], but more work is needed to better understand the socio-economic and behavioural determinants [10–12, 20]. Although COVID-19 has had a disproportionate impact on older adults and minority groups [3–8, 10–14], there is limited research on racial and ethnic differences in COVID-19 diagnosis and preventive behaviours for older populations. To the best of our knowledge, this study is the first to use a prospective national sample of older adults to examine the relationships between race and ethnicity, COVID-19 diagnosis, and adherence to COVID-19 mitigation behaviours. Assessing infection and behavioural adherence across multiple racial and ethnic groups in one data set allows us to explore these relationships more comprehensively. Furthermore, the use of a national sample and survey sampling weights enables our results to be generalisable for the country. Although COVID-19 mitigation behaviour policies have varied by state, our study focuses on three core behaviours recommended by the CDC: handwashing, masking, and social distancing. Therefore, the purpose of this study was to 1) examine racial and ethnic differences in COVID-19 diagnosis among older adults and 2) examine racial and ethnic differences in adherence to COVID-19 mitigation behaviours (handwashing, masking, and social distancing) among older adults.

## Methods

### Data source

We analysed data from the National Health and Aging Trends Study (NHATS), a longitudinal panel study that uses a nationally representative U.S. sample of Medicare beneficiaries aged 65 or older. We specifically used the 2019 wave and 2020 NHATS COVID-19 supplement, a self-administered survey mailed out between June and October 2020. The majority of the responses were completed in July 2020 (51.01%) or August 2020 (33.27%) [25]. Overall, the response rate for the supplement was 82.23%, leading to a final sample of 3257 older adults [25]. All other non-COVID-19 variables were retrieved from the 2019 NHATS wave.

### COVID-19 diagnosis

The key dependent variable, self-reported COVID-19 diagnosis, was determined via two questions from the NHATS COVID-19 supplement. Respondents were first asked, ‘Has a doctor or other health professional told you that you may have had COVID-19?’, and possible responses were ‘Yes, definitely’, ‘Yes, possibly’, and ‘No’. Then, respondents were asked, ‘Have you had a positive test for COVID-19?’ and they could answer either ‘Yes’ or ‘No’. We defined a positive COVID-19 diagnosis as a ‘Yes, definitely’ or ‘Yes, possibly’ answer from a health professional or a ‘Yes’ from a COVID-19 diagnostic test. A sensitivity analysis was also conducted in which ‘Yes, possibly’ was recoded into a ‘No’ diagnosis; however, the study conclusions remained the same (Supplementary Table 1).

### COVID-19 mitigation behaviours

Our other dependent variables were the three main COVID-19 mitigation behaviours – handwashing, masking, and social distancing. All three behaviours were asked as a part of the question, ‘During the COVID-19 outbreak, have you ever done the following to keep the disease from spreading?’. Handwashing was measured as ‘Frequently wash your hands or use sanitiser’, and the available answers were ‘Yes’ or ‘No’. Masking was measured as ‘Wear a face mask when going out’ and could be answered as ‘Yes’, ‘No’, or ‘Does not apply’. Social distancing was measured as ‘Stay at least 6 feet away from people not living with you’ and had the potential answers of ‘Yes’, ‘No’, or ‘Does not apply’. Any ‘Does not apply’ response was coded as missing. For several analyses, we aggregated the number of mitigation behaviours adhered to in order to construct a composite score, with a range from 0 (practised no behaviours) to 3 (practised all three behaviours).

### Race and ethnicity

Self-reported race and ethnicity included non-Hispanic White (hereafter, White), non-Hispanic Black (hereafter, Black), Hispanic, Asian, and Other. The Other category (*n* = 71) merged several racial and ethnic groups due to small sample sizes and to retain the full study sample for statistical analyses. Other included American Indian (28.17%, *n* = 20), multiracial (2.82%, *n* = 2), and unspecified (69.01%, *n* = 49).

### Covariates

The regression models adjusted for a large number of sociodemographic and health covariates identified as social determinants linked to COVID-19 health and well-being that we had available in the NHATS data [26]. Sociodemographic covariates were age, gender (female or male), highest level of education (less than high school, high school, or college), total income, marital status (married or unmarried), total number of people in the household, metropolitan area residence (metro or non-metro), and type of residential setting (in the community or in residential care or nursing home).

Health covariates included self-rated overall health (poor, fair, good, very good, or excellent recorded on a 0–4 scale), body mass index (BMI), activities of daily living (ADL) limitations (no ADLs or at least one ADL), use of a proxy respondent, and a number of common COVID-19 comorbidities – major depressive disorder, generalised anxiety disorder, history of heart attack, history of hypertension, history of diabetes, and history of stroke.

### Analysis plan

For the dependent variable of COVID-19 diagnosis, we analysed racial and ethnic differences in the odds of COVID-19 diagnosis using a series of multiple logistic regression models that were initially unadjusted (Model A) and then sequentially adjusted for sociodemographics (Model B), health (Model C), and COVID-19 mitigation behaviours (Model D). There were statistically significant correlations between all three behaviours, and the model automatically omitted some practices due to multicollinearity. Therefore, individual mitigation behaviours were combined into one composite score for Model D. The highest individual variance inflation factor (VIF) was 1.87 and the average VIF was 1.23, which indicates that there is no harmful multicollinearity.

For the dependent variables of adherence to COVID-19 mitigation behaviours (handwashing, masking, and social distancing), three separate logistic regression models were constructed for each practice. All models were adjusted for sociodemographics and health. The average VIF was 1.24 for all three models, which also indicates that there was no harmful multicollinearity.

To maximise the full sample size and minimise bias due to missing data (10–15% depending on the dependent variable), multiple imputation by chained equations (MICE) generated 100 imputed data files with 10 iterations each. There were no substantial differences in results generated from MICE compared with list-wise deletion for both research questions. All models applied complex survey sampling weights. Statistical analyses were performed in Stata statistical software version 17 (StataCorp LLC, College Station, TX, USA) with two-tailed tests at a 0.05 significance level.

### Ethics approval

This study was approved by the SUNY Upstate Institutional Review Board for the Protection of Human Subjects and exempted from review due to the use of a limited data set (#1760882-1).

## Results

### Sample characteristics

Of the 3257 sample respondents, 3.1% (*n* = 98) reported a positive COVID-19 diagnosis in 2020. The majority of cases were identified by a COVID-19 test alone (53.1%, *n* = 52), while less came from a health professional’s diagnosis alone (20.4%, *n* = 20) or from both a health professional and a test (26.5%, *n* = 26). As seen in Table 1, the average age in years for respondents was 74.2 (*SD* = 6.6). Over half (57.9%) were female, and the most common level of education was a high school degree or equivalent, but not a college degree (48.2%). The mean income was approximately $61090 (*SD* = $67350). The percentage of respondents who were married was slightly less than half (49.2%), and the average household size was 1.9 persons (*SD* = 1.0). About 80.1% lived in a metropolitan area, while 6.9% lived in residential care or a nursing home. Respondents rated their own health on average as 2.3 (*SD* = 0.98), which is between a ‘good’ (2) and ‘very good’ (3) rating for health. The average BMI was 27.9 (*SD* = 6.1), indicating that the majority were overweight. At least one ADL limitation was reported by 15.8% of respondents, and 2.2% of the surveys were completed by a proxy. Hypertension (73.9%) and diabetes (28.1%) were the most common health conditions reported.

**Table 1.** Sample characteristics

	Whole sample (*n* = 3257)	White (*n* = 2472)	Black (*n* = 543)	Hispanic (*n* = 133)	Asian (*n* = 38)	Other (*n* = 71)	Bivariate test^a^
Age (mean, SD)	74.2 (6.6)	74.2 (6.6)	74.0 (6.3)	73.5 (6.5)	73.3 (6.0)	75.9 (7.8)	*F*(4,3252) = 1.9, *P* = 0.11
Female (%, *n*)	57.9% (1887)	56.6% (1398)	65.6% (356)	55.6% (74)	42.1% (16)	60.6% (43)	χ^2^(4) = 19.3, *P* < 0.01
Highest level of education							χ^2^(8) = 306.9, *P* < 0.001
<High school	14.6% (471)	9.8% (243)	29.4% (159)	48.1% (64)	0.00% (0)	13.9% (5)	
High school	48.2% (1552)	49.2% (1216)	46.7% (252)	39.1% (52)	26.3% (10)	61.1% (22)	
College	37.1% (1195)	41.0% (1012)	23.9% (129)	12.8% (17)	73.7% (28)	25.0% (9)	
Income (thousands)	61.1 (67.4)	68.3 (70.6)	36.2 (43.6)	27.4 (25.6)	98.5 (118.2)	43.7 (44.3)	*F*(4,3236) = 91.6, *P* < 0.001
Marital status							χ^2^(4) = 101.8, *P* < 0.001
Not married	50.8% (1655)	46.7% (1153)	69.7% (378)	57.9% (77)	34.2% (13)	47.9% (34)	
Married	49.2% (1600)	53.3% (1318)	30.3% (164)	42.1% (56)	65.8% (25)	52.1% (37)	
Household size	1.93 (1.01)	1.84 (0.88)	2.19 (1.32)	2.38 (1.28)	2.32 (1.28)	2.04 (1.20)	*F*(4,3250) = 22.3, *P* < 0.001
Metropolitan residence	80.1% (2610)	77.0% (1904)	90.1% (489)	92.5% (123)	97.4% (37)	80.3% (57)	χ^2^(4) = 68.4, *P* < 0.001
Residential setting							χ^2^(4) = 12.6, *P* = 0.01
Community-dwelling	93.1% (3033)	92.3% (2281)	96.1% (522)	96.2% (128)	94.7% (36)	93.0% (66)	
Residential care or nursing home	6.9% (224)	7.7% (191)	3.9% (21)	3.8% (5)	5.3% (2)	7.0% (5)	
Self-rated health (0–4: poor–excellent)	2.28 (0.98)	2.39 (0.96)	1.95 (0.93)	1.72 (1.03)	2.26 (1.03)	2.18 (1.07)	*F*(4,3248) = 35.6, *P* < 0.001
Body mass index	27.9 (6.1)	27.6 (6.0)	29.3 (6.6)	28.5 (5.6)	24.8 (3.3)	28.1 (6.3)	*F*(4,3186) = 10.8, *P* < 0.001
ADL limitations							χ^2^(4) = 28.6, *P* < .001
None	84.2% (2728)	85.7% (2108)	78.6% (425)	82.2% (106)	97.4% (37)	73.2% (52)	
At least one	15.8% (512)	14.3% (353)	21.4% (116)	17.8% (23)	2.6% (1)	26.8% (19)	
Proxy respondent	2.2% (72)	1.9% (46)	3.5% (19)	2.3% (3)	2.6% (1)	4.2% (3)	χ^2^(4) = 6.9, *P* = 0.14
Depression	8.9% (288)	6.9% (170)	13.5% (73)	25.8% (34)	10.5% (4)	10.0% (7)	χ^2^(4) = 72.6, *P* < 0.001
Anxiety	7.7% (248)	6.8% (168)	9.8% (53)	15.0% (20)	0.0% (0)	9.9% (7)	χ^2^(4) = 19.6, *P* < 0.01
Dementia	14.9% (485)	13.4% (332)	19.0% (103)	21.8% (29)	2.6% (1)	28.2% (20)	χ^2^(4) = 30.7, *P* < 0.001
History of heart attack	16.9% (548)	16.5% (406)	16.9% (91)	21.2% (28)	5.4% (2)	30.0% (21)	χ^2^(4) = 14.0, *P* < 0.01
History of hypertension	73.9% (2401)	70.8% (1745)	87.7% (476)	79.7% (106)	71.1% (27)	66.2% (47)	χ^2^(4) = 70.3, *P* < 0.001
History of diabetes	28.1% (910)	23.4% (576)	43.1% (233)	51.9% (69)	35.1% (13)	26.8% (19)	χ^2^(4) = 124.6, *P* < 0.001
History of stroke	12.4% (402)	12.1% (298)	13.9% (75)	9.8% (13)	8.1% (3)	19.1% (13)	χ^2^(4) = 5.5, *P* = 0.24

a Chi-square for categorical variables and ANOVA for continuous variables.

### Bivariate results

#### COVID-19 diagnosis

As shown in Table 2, COVID-19 diagnosis was significantly associated with race and ethnicity (χ^2^(4) = 14.0, *p* < 0.01). Positive COVID-19 diagnoses were notably highest among Hispanic (8.5%) and Black (3.4%) older adults compared with all racial and ethnic groups in the whole sample (3.1%).

**Table 2.** COVID-19 diagnosis and adherence to COVID-19 mitigation behaviours by race and ethnicity

	Whole sample (*n* = 3257)	White (*n* = 2472)	Black (*n* = 543)	Hispanic (*n* = 133)	Asian (*n* = 38)	Other (*n* = 71)	Bivariate test^a^
COVID-19 diagnosis							
Negative	96.9% (3091)	97.3% (2362)	96.6% (506)	91.5% (119)	97.3% (36)	97.1% (68)	χ^2^(4) = 14.0, *P* < 0.01
Positive	3.1% (98)	2.7% (66)	3.4% (18)	8.5% (11)	2.7% (1)	2.9% (2)	
Adherence to COVID-19 mitigation behaviours							
Handwashing	97.1% (3087)	96.6% (2327)	98.7% (525)	99.2% (130)	100.0% (37)	97.1% (68)	χ^2^(4) = 9.9, *P* = 0.04
Masking	96.6% (3020)	95.8% (2263)	99.2% (523)	100.0% (130)	100.0% (37)	98.5% (67)	χ^2^(4) = 23.1, *P* < 0.001
Social distancing	92.2% (2841)	91.0% (2127)	95.9% (495)	96.8% (122)	100.0% (37)	90.9% (60)	χ^2^(4) = 21.5, *P* < 0.001
All practices (0–3) (mean, SD)	2.87 (0.42)	2.84 (0.46)	2.94 (0.27)	2.96 (0.20)	3.00 (0.00)	2.85 (0.40)	*F* (4,3002) = 8.1, *P* < 0.001

a Chi-square for categorical variables and ANOVA for continuous variables.

#### COVID-19 mitigation behaviours

Handwashing, masking, and social distancing were all significantly associated with race and ethnicity (Table 2). The composite score aggregating these practices was also significantly associated with race and ethnicity (*F*(4,3002) = 8.1, *p* < 0.001). Adherence to all three behaviours was higher among Asians (mean = 3.00; *SD* = 0.0), Hispanics (mean = 2.96, *SD* = 0.20), and Blacks (mean = 2.94, *SD* = 0.27) compared with the whole sample of older adults (mean = 2.87, *SD* = 0.42).

### Multiple logistic regression results

#### COVID-19 diagnosis

In Table 3, a series of logistic regression models were developed to examine racial and ethnic differences in the odds of COVID-19 diagnosis. Model A was unadjusted, Model B was adjusted for sociodemographics, Model C was further adjusted for health, and the final model, Model D, was further adjusted for COVID-19 mitigation behaviours. In every model, Black, Hispanic, and Asian older adults all had elevated odds of COVID-19 compared with White older adults as the reference group. In Model D, for example, the odds for COVID-19 were 1.13 times higher for Blacks (aOR = 1.13, 95% CI: 0.55, 2.30, *p* = 0.73), 2.71 times higher for Hispanics (aOR = 2.71, 95% CI: 1.20, 6.12, *p* < 0.01), and 1.68 times higher for Asians (aOR = 1.68, 95% CI: 0.22, 12.88, *p* = 0.61). Respondents of Other race and ethnicity, however, had lower odds of COVID-19 diagnosis, with the odds ratio dropping to 0.79 (aOR = 0.79, 95% CI: 0.14, 4.34, *p* = 0.78) in Model D.

**Table 3.** Unadjusted and adjusted odds of positive COVID-19 diagnosis by race and ethnicity

	Model A OR (95% CI), *P*	Model B aOR (95% CI), *P*	Model C aOR (95% CI), *P*	Model D aOR (95% CI), *P*
White	Reference	Reference	Reference	Reference
Black	1.47 (0.78–2.77), 0.22	1.19 (0.61–2.29), 0.61	1.14 (0.57–2.28), 0.70	1.13 (0.55–2.30), 0.73
Hispanic	3.82 (1.79–8.14), <0.01	2.67 (1.22–5.84), 0.02	2.75 (1.23–6.15), 0.02	2.71 (1.20–6.12), <0.01
Asian	1.83 (0.25–13.53), 0.55	1.18 (0.17–8.14), 0.86	1.72 (0.22–13.58), 0.60	1.68 (0.22–12.88), 0.61
Other	1.06 (0.23–4.85), 0.94	0.89 (0.19–4.16), 0.88	0.80 (0.15–4.36), 0.79	0.79 (0.14–4.34), 0.78
Model significance	*F*(4,53) = 3.36, *P* = 0.02	*F*(13,53) = 4.47, *P* < 0.001	*F*(23,53) = 9.43, *P* < 0.001	*F*(25,53) = 11.08, *P* < 0.001

*Note.* Model A is an unadjusted crude model, Model B is adjusted for sociodemographics, Model C is adjusted for sociodemographics and health, and Model D is adjusted for sociodemographics, health, and COVID-19 mitigation behaviours.

Although the odds for COVID-19 were elevated for all older adults of colour, higher odds for COVID-19 were statistically significant for only Hispanic older adults in all models. For the unadjusted crude model, Hispanic older adults had 3.82 times higher odds of COVID-19 (OR = 3.82, 95% CI: 1.79, 8.14, *p* < 0.01) compared with White older adults. This relationship remained statistically significant after adjusting for sociodemographics (aOR = 2.67, 95% CI: 1.22, 5.84, *p* = 0.02), health (aOR = 2.75, 95% CI: 1.23, 6.15, *p* = 0.02), and COVID-19 mitigation behaviours (aOR = 2.71, 95% CI: 1.20, 6.12, *p* < 0.01).

#### COVID-19 mitigation behaviours

Compared to White older adults, all other racial and ethnic groups generally had higher odds of handwashing, masking, and social distancing (Table 4). Black older adults had 7.94 times higher odds of masking (aOR = 7.94, 95% CI: 2.33, 27.04, *p* < 0.01) and 2.33 times significantly higher odds of social distancing (aOR = 2.33, 95% CI: 1.28, 4.24, *p* < 0.01) after adjusting for sociodemographics and health. All Asian respondents, meanwhile, practised all three behaviours and all Hispanic respondents practised masking, which prevented the calculation of an odds ratio due to no variation. When Hispanic and Asian older adults were combined with those of Other race and ethnicity, however, the odds of masking were 18.13 times significantly higher than White older adults (aOR = 18.13, 95% CI: 1.90, 173.21, *p* = 0.01).

**Table 4.** Adjusted odds of adherence to COVID-19 mitigation behaviours by race and ethnicity

	Handwashing aOR (95% CI), *P*	Masking aOR (95% CI), *P*	Social distancing aOR (95% CI), *P*
White	Reference	Reference	Reference
Black	2.18 (0.81–5.91), 0.12	7.94 (2.33–27.04), <0.01	2.33 (1.28–4.24), 0.01
Hispanic	6.53 (0.80–53.21), 0.08	NA	2.41 (0.79–7.29), 0.12
Asian	NA	NA	NA
Other	4.00 (0.77–20.83), 0.10	18.13 (1.90–173.21), 0.01	1.22 (0.44–3.35), 0.70
			
Model significance	*F*(23,53) = 4.46, *P* < 0.001	*F*(22,52) = 7.46, *P* < 0.001	*F*(23,52) = 3.29, *P* < 0.001

*Note.* NA, not available, merged with ‘Other’ race and ethnicity.

## Discussion

### COVID-19 diagnosis

This study investigated the relationships between race and ethnicity, COVID-19 diagnosis, and adherence to COVID-19 mitigation behaviours among older U.S. adults. For our first aim on racial and ethnic differences in the odds of a positive COVID-19 diagnosis, we found elevated odds, though not statistically significant, for the majority of the racial and ethnic minority older adults in our sample. Higher odds of COVID-19 among minority individuals have been shown in other data as well [3, 10–12]; differences in odds and statistical significance may tie into the older age of our sample and their correspondingly lower likelihood of working frontline jobs. Varying adjusted odds may additionally come from different covariates used in analyses to represent structural inequities.

Hispanic older adults in particular were the only group to have significantly higher odds for COVID-19 compared with White older adults, even after adjusting for sociodemographics, health, and COVID-19 mitigation behaviours. The initial unadjusted model indicates 3.82 times higher odds for COVID-19 among Hispanic older adults compared with Whites, and this remained high at 2.71 times higher odds in our fully adjusted final model. The elevated odds for Hispanic older adults may be partially tied to Hispanic individuals having the lowest average income and the largest household size across all racial and ethnic groups. Income has been previously referenced as a likely factor in COVID-19 diagnosis for older people of colour [8]. It is associated with lower quality and more crowded living environments, and it can influence older adults to take more high-risk work positions that are less likely to have remote options [8]. Low income can also make it more difficult to acquire resources such as hand sanitisers, especially as prices for cleaning products surged in the early months of the pandemic, and income may be related to less opportunities and infrastructure for washing one’s hands [18]. Larger, multigenerational households have been previously noted as a potential COVID-19 risk factor [8, 20, 27]. In the United Kingdom, one study found that every additional person in a household increased the odds of a positive PCR COVID-19 test by 6% [27].

### COVID-19 mitigation behaviours

For our second aim, we found that Black and Hispanic older adults have greater odds of adhering to the COVID-19 mitigation behaviours in the regression models. Hispanic older adults had 100% adherence to masking, and all Asian respondents had 100% adherence to all three behaviours, which prevented the calculation of an odds ratio for these groups. These results overall match the current literature, which has found high adherence to handwashing and social distancing among Asian Americans [18, 20], the greater likelihoods of Black and Hispanic adults to wash their hands compared with Whites [21], the higher odds of Hispanic individuals to social distance compared with White individuals [21], and the greater probabilities for Asian, Black, and Hispanic Americans to wear masks than Whites [22].

Because racial and ethnic minority older adults had higher odds of COVID-19 than White older adults, it has been especially critical for them to take precautionary measures against the disease. Furthermore, the significantly higher prevalence of masking and social distancing we found for minority older adults in particular is reassuring, as recent evidence suggests that these two mitigation behaviours are more important than handwashing in reducing the spread of SARS-CoV-2 [28]. However, the same vulnerability requiring older adults of colour to work harder to protect themselves is itself another indicator of the disparities and burden on these populations. One potential explanation for differences in adherence to handwashing between racial and ethnic groups may be varying levels of concern over being infected with SARS-CoV-2 [18]. Older adults of colour can have less social and economic safety nets than their White counterparts, and they may fear the consequences of COVID-19 more as a result [21]. This can be compounded in certain populations with longstanding distrust of medical institutions and low access to quality health care, leading to a general reliance on COVID-19 mitigation behaviours [20].

It is important to emphasise that the disparities discussed above are the results of social determinants of health. Sociocultural factors may account for some of the differences between racial and ethnic groups; for example, Asian respondents’ high rates of masking could be related to cultural norms in favour of masking in East Asian and Southeast Asian nations [29, 30]. While masks can have deep social, political, and environmental importance for specific Asian American populations, head and face coverings are more stigmatised for other U.S. demographics [29, 30]. This might have contributed to the different rates of masking adherence between Asian older adults and the White comparison group. Perhaps more consequential, however, is the problem of structural racism [26]. Issues such as low-income and high-density housing do not reflect personal choices on the part of the respondents, but rather inequities in social and economic resources due to discrimination that has limited the educational, residential, and economic options for people of colour over generations [8, 10, 21]. Equitably addressing COVID-19 will thus involve not only traditional public health strategies but also systemic approaches that acknowledge and alleviate the barriers to well-being for minority populations.

### Implications

Despite higher rates of handwashing, masking, and social distancing, older minority respondents reported greater percentages of COVID-19 diagnoses. Causation cannot be inferred from these findings, but they may suggest that COVID-19 is a greater problem than any individual can wholly take on alone, especially among demographics already at structural disadvantage. Reducing COVID-19 morbidity and mortality in future waves will plausibly require action at higher socio-ecological levels, especially in the realms of policy, health care, and community action [31, 32]. As COVID-19 hospitalisations and deaths threaten to rise again, effective and timely large-scale action could be critical to preventing the virus’ spread [2, 31]. That said, mitigation behaviours such as masking, social distancing, and vaccination, the last not yet publicly available at the time of data collection, are still important tools shown to reduce infection risk in other studies [33–35]. Comprehensive COVID-19 mitigation will likely include these behaviours to supplement broader contextual factors to minimise disparities in COVID-19 infection.

### Strengths and limitations

There were four limitations for our study. First, data were only available during the early stages of the pandemic in 2020, and it is possible that the number of COVID-19 cases was underestimated, which may be due to the limited availability of diagnostic tests and relatively high older adult mortality [3]. This may be especially true in under-resourced communities with limited access to healthcare or diagnostic tests. Based on available CDC data, however, the per cent of COVID-19-positive diagnoses we observed generally appears to be in line with the weekly incidence rate during our study window [2]. Second, the responses for handwashing, masking, and social distancing adherence were binary, which measures neither the frequency of performing each practice nor how it may vary across settings. Third, except for those who self-identified as White, Black, or Hispanic, there was a relatively low number of respondents from other racial and ethnic groups. This contributed to low statistical power in several analyses, and multiple groups were merged with ‘Other’ race and ethnicity to proceed with statistical analyses. Furthermore, potential confounding may be present due to differences in sociodemographic and health characteristics across racial and ethnic groups, but small sample sizes in several groups precluded approaches such as propensity score matching to minimise selection bias. Fourth, similar to other large national surveys, most responses were self-reported, including all COVID-19 variables. Though self-reported data may contribute to inaccuracies due to recall bias, we believe that this impact may be minimal since the first COVID-19 case was in early 2020 and most surveys were completed in July 2020. Despite these limitations, to our knowledge, the findings in our study present the first examination of the relationship between COVID-19 diagnosis and adherence to COVID-19 mitigation behaviours using a nationally representative U.S. older adult sample with multiple racial and ethnic groups.

## Conclusions

Our study identified higher positive COVID-19 diagnoses among racial and ethnic minority older adults, and COVID-19 diagnoses were significantly higher for Hispanic older adults. Faced with the emerging threat of more infectious SARS-CoV-2 strains, protecting older adults from COVID-19 is as important as ever. It will be especially critical to focus on racial and ethnic minority communities where adherence to COVID-19 mitigation behaviours has not been enough to overcome pre-existing structural inequities in health, health care, and socio-economic status. Given our available data limiting temporal analyses, future research should analyse how behaviours may have evolved over time since the summer of 2020, especially considering more recent waves and strains of the virus.

## Supporting information

Wong and Lovier supplementary material Wong and Lovier supplementary material

## Data Availability

This study uses non‐public sensitive data, which may be obtained through an application from the National Health and Aging Trends Study (https://nhats.org/).

## Supplementary material

For supplementary material accompanying this paper visit http://doi.org/10.1017/S0950268823001607. click here to view supplementary material
