# Supplementary material for: Racial and ethnic disparities in COVID-19 diagnosis and adherence to mitigation behaviours in a national United States older adult sample
Source: Epidemiol Infect. 2023 Oct 6;151:e175. doi: 10.1017/S0950268823001607 (PMC10600897; doi:10.1017/S0950268823001607)
Supplement: Wong and Lovier supplementary material [file S0950268823001607sup001.docx]

# Supplementary Table 1. Unadjusted and Adjusted Odds of Positive COVID-19 Diagnosis by Race and Ethnicity

|  | Model A  OR (95% CI), *P* | Model B  aOR (95% CI), *P* | Model C  aOR (95% CI), *P* | Model D  aOR (95% CI), *P* |
| --- | --- | --- | --- | --- |
| White | reference | reference | reference | reference |
| Black | 1.76 (0.95–3.27), .07 | 1.20 (0.59–2.38), .59 | 1.09 (0.53–2.27), .81 | 1.16 (0.55–2.45), .68 |
| Hispanic | 4.44 (2.01–9.81), <.01 | 2.55 (1.18–5.55), .02 | 2.99 (1.37–6.50), <.01 | 2.89 (1.40–5.94), <.01 |
| Asian | 2.42 (0.33–17.78), .38 | 1.63 (0.23–11.43), .62 | 2.46 (0.32–18.93), .38 | 2.22 (0.29–16.97),  .43 |
| Other | 1.40 (0.30–6.51), .67 | 2.87 (0.61–13.54), .88 | 2.13 (0.25–18.10), .48 | 2.80 (0.29–26.75), .36 |
|  |  |  |  |  |
| Model Significance | F(4, 52) = 4.02,  *P* = .01 | F(13, 43) = 7.03,  *P* < .001 | F(23, 33) = 6.12,  *P* < .001 | F(25, 31) = 5.09,  *P* < .001 |

*Note.* COVID-19 diagnosis based on a COVID-19 test or a “Yes, definitely” from a doctor. Model A is an unadjusted crude model, Model B is adjusted for sociodemographics, Model C is adjusted for sociodemographics and health, and Model D is adjusted for sociodemographics, health, and COVID-19 mitigation behaviors.
